# Supercritical fluid extract of *Lycium chinense* Miller root inhibition of melanin production and its potential mechanisms of action

**DOI:** 10.1186/1472-6882-14-208

**Published:** 2014-06-28

**Authors:** Huey-Chun Huang, Wen-Ying Huang, Tsang-Chi Tsai, Wan-Yu Hsieh, Wang-Ping Ko, Kuei-Jen Chang, Tsong-Min Chang

**Affiliations:** 1 Department of Medical Laboratory Science and Biotechnology, China Medical University, Taichung, Taiwan; 2 Department of Applied Cosmetology & Master Program of Cosmetic Sciences, HungKuang University, No. 1018, Sec. 6, Taiwan Boulevard, Shalu Dist, Taichung City 43302, Taiwan; 3 O’right Plant Extract R&D Center, Taoyuan, Taiwan

**Keywords:** *Lycium chinense* Miller, melanogenesis, MAPK, PKA, ROS

## Abstract

**Background:**

The mode of action of *Lycium chinense* Miller root extract in skin care has never been explored. In the present study, *Lycium chinense* Miller root was extracted by the supercritical fluid CO_2_ extraction method.

**Methods:**

In the present study, the components of the root extract were analyzed by HPLC. The effects of the extract on tyrosinase activity and melanin content were determined spectrophotometrically; the expression of melanogenesis-related proteins was determined by Western blotting; the possible signaling pathways involved in the root extract-mediated depigmentation were also investigated using specific inhibitors.

**Results:**

The results revealed that the SFE of *Lycium chinense* Miller root (2.37-7.11 mg/mL) effectively suppressed intracellular tyrosinase activity and decreased the melanin content in B16F10 cells. The root extract also effectively decreased intracellular reactive oxygen species (ROS) levels. Furthermore, the root extract decreased the expression of melanocortin 1 receptor (MC1R), microphthalmia-associated transcription factor (MITF), tyrosinase and tyrosinase-related protein-1 (TRP-1) and then inhibited melanogenesis in B16F10 cells. The root extract also showed antioxidant capacities and depleted cellular ROS.

**Conclusions:**

Our results indicate that the SFE of *Lycium chinense* Miller root inhibited melanogenesis in B16F10 cells by down-regulation of both mitogen-activated protein kinases (MAPK) and protein kinase A (PKA) signaling pathways or through its antioxidant properties.

## Background

Melanin is secreted by melanocytes that are distributed in the basal layer of the skin epidermis [1]. Melanin is responsible for skin color and also plays a key role in protecting the skin against ultraviolet (UV) sunlight damage. Various dermatological disorders result from the accumulation of an excessive level of epidermal melanin. Hyperpigmented skin disorders include melasma, age spots, freckles and sites of actinic damage [2]. The inhibitors of melanogenesis have been increasingly applied in skin care products for the treatment or prevention of skin hyperpigmentation [3]. Tyrosinase (EC 1.14.18.1) is a copper-containing enzyme that catalyzes the first two steps of melanin synthesis. It first hydroxylates L-tyrosine to L-3,4-dihydroxyphenylalanine (L-DOPA), and L-DOPA is further oxidized to the corresponding *o*-dopaquinone [4]. There are many factors that participate in the regulation of melanin synthesis. For example, the microphthalmia-associated transcription factor (MITF), tyrosinase-related protein-1 (TRP-1) and tyrosinase-related protein-2 (TRP-2) are known to regulate the production of melanin [5-7]. Moreover, the melanocortin 1 receptor (MC1R) also plays an important role in melanocyte stimulating hormone (MSH)-induced melanogenesis [8]. Furthermore, it is reported that melanogenesis produces hydrogen peroxide (H_2_O_2_) and reactive oxygen species (ROS), which lead to the generation of high-grade oxidative stress in melanocytes. In particular, certain ROS scavengers and inhibitors of ROS generation have been reported to inhibit UV-induced melanogenesis [9]. Antioxidants such as reduced glutathione (GSH) and ascorbic derivatives have been applied to inhibit or delay hyperpigmentation [10,11]. Hence, antioxidants and free radical scavengers also play an important role in the regulation of melanin synthesis. Recently, we also reported that certain plant extracts [12], essential oils [13,14] and microbial metabolites [15,16] exhibit dual antioxidant and anti-melanogenic activities.

*Lycium chinense* Miller, also called boxthorn, is a plant belonging to the family Solanaceae that is widely distributed in East Asia. The leaves and fruits of boxthorn have been used as foods or medicine in the Orient. Boxthorn leaves have been reported to exhibit tranquillizing, thirst-quenching and anti-aging activity. In addition, the leaves of *Lycium chinense* Miller are known to reduce the risk of certain diseases such as arteriosclerosis, diabetes and night blindness [17]. The fruits of *Lycium chinense* Miller have been used traditionally for anti-aging [18] and hepatoprotective purposes [19]. In addition, the fruits have been reported to show antipyretic, hypoglycemic and hypotensive activities in animal models [20]. Recently, it was reported that zeaxanthin dipalmitate, a carotenoid from *L. chinense* fruits, significantly reduced the proliferation of myofibroblast-like cells (MFBLCs) and collagen synthesis in cultured hematopoietic stem cells (HSCs) *in vitro*[21]. However, there is relatively little knowledge regarding the modes of action of *Lycium chinense* Miller root extract in skin care or dermatology.

The aim of current study was to investigate the antimelanogenic activity of the supercritical fluid extract of *Lycium chinense* Miller root in murine B16F10 melanoma cells. We also evaluated the potential action mechanisms of the root extract in melanogenesis.

## Methods

### Chemicals and reagents

The chemical reagents were purchased from Sigma Chemical Co. (St. Louis, MO, USA). The antibodies were obtained from Santa Cruz Biotech (Santa Cruz, CA, USA) and the ECL reagent from Millipore (MA, USA). Protein kinase regulators, including3-isobutyl-1-methyl-xanthine (IBMX), SB203580 (p38 MAPK-inhibitor), SP600125 (c-Jun N-terminal kinase inhibitor; JNK inhibitor) and PD98059 (MEK 1/2-inhibitor), were obtained from Tocris (Ellisville, Missouri, USA).

### Preparation of Lycium chinense Miller root powder

The *Lycium chinense* Miller roots were harvested in June 2012 from a farm located at Guanyin Township, Taoyung County, Taiwan. The roots of *Lycium chinense* Miller were identified in the National Research Institute of Chinese Medicine (NRICM), Ministry of Health and Welfare, Taiwan. Besides, there was a botanically identified voucher specimen (NHP-00219) deposited in the institute. The roots were washed completely, exposed to sunlight and air-dried for one day. The roots were sliced into pieces and exposed to sunlight for 7 more days and then dried at 80°C for 2 h in an oven. The dehydrated root slices were pulverized to a fine powder (#20 mesh) with a centrifugal mill (Retsch Ultra Centrifugal Mill and Sieving Machine, Type ZM1, Haan, Germany). The powder was collected in a sealed glass bottle and stored at 25°C until use.

### Supercritical fluid CO_2_ extraction (SFE) of *Lycium chinense* Miller root

The pulverized, desiccated *Lycium chinense* Miller root (83 g) was placed in the extraction vessel (200 ml) of a supercritical fluid CO_2_ extraction (SFE) apparatus (SFE-400S-2000, Metal Industries Research & Development Centre; MIRDC; Kaohsiung, Taiwan). Extraction was performed with a 10% co-solvent of ethanol in supercritical fluid CO_2_ (flow rate, 5.0 ml/min) at 5,000 psi (=350 bar) at 50°C for 2 h. The extracts were evaporated to dryness in a rotary evaporator at 40°C under reduced pressure. The concentrated SFEs were weighed and stored at -20°C. In the following experiments, the SFEs were re-dissolved in dimethyl sulfoxide (DMSO) as indicated.

### HPLC analysis of *Lycium chinense* Miller root SFE

The *Lycium chinense* Miller root SFE sample was mixed with an internal standard solution (66 mg of naringin was diluted to 12 ml with 70% methanol) in a ratio of 99:1. Then, samples were spiked with various concentrations of stock solutions and analyzed. A stock solution was prepared by dissolving two marker substances (rutin, 1 mg and liquiritigenin, 2 mg in 1 ml of 70% methanol) and stored in a refrigerator. Before adding the internal standard solution, the stock solution was then diluted with 70% methanol into a series of standard solutions (rutin: 4.95, 6.6, 9.9, 19.8 and 39.6 μg/ml; liquiritigenin: 7.07, 8.25, 9.9, 12.375 and 24.75 μg/ml). Twenty microliters of these solutions was injected, and the samples were analyzed twice by the HPLC method; standard curves were plotted according to the peak areas versus concentrations. Recovery was determined by comparing of the amount of marker substances added with the marker substances found. The limits of detection were based on a signal-to-noise (S/N) ratio of 3:1 as a minimum.

HPLC was performed on an Agilent 1220 series system. Satisfactory separation of the market substances, obtained with a reversed-phase column (Cosmosil 5 C18-AR II, 5 μm, 25 cm × 4.6 mm I.D., Nacalai Tesque, Kyoto, Japan) at 25°C, was eluted at a flow rate of 0.8 ml/min with a linear solvent gradient of A-B [A = 0.5% CH_3_COOH; B = CH_3_CN: CH_3_OH = 2: 1 (v/v)] as follows: 5 min, 0% B; 10 min, 15% B; 20 min, 20% B; 50 min, 26% B; 70 min, 30% B.

### Cell culture and cell viability assay

B16F10 cells (ATCC CRL-6475, BCRC60031) were obtained from the Bioresource Collection and Research Center (BCRC), Taiwan. The cells were maintained in DMEM (Hyclone, Logan, UT) supplemented with 10% fetal bovine serum and 1% antibiotics at 37°C, 5% CO_2_ in a humidified incubator.

The cell viability assay was performed using 3-(4, 5-dimethylthiazol-2-yl)-2,5-diphenyltetrazolium bromide (MTT) [22]. The cells were exposed to various concentrations of *Lycium chinense* Miller root SFE for 24 h, and the MTT solution was then added to the wells. The insoluble derivative of MTT produced by intracellular dehydrogenase was solubilized with ethanol-DMSO (1:1 mixture solution). The absorbance of the wells at 570 nm was read using a microplate reader.

### Assay of mushroom tyrosinase activity

Enzyme inhibition experiments were conducted as previously described [23]. Briefly, 10 μL of an aqueous solution of mushroom tyrosinase (200 units) was added to a 96-well microplate to produce a 200-μL mixture containing 5 mM L-DOPA, which was dissolved in 50 mM phosphate buffered saline (PBS) (pH 6.8), *Lycium chinense* Miller root SFE (5.93, 11.85 and 23.7 mg/mL) or kojic acid (200 μM). The assay mixture was incubated at 37°C for 30 min, and the absorbance of dopachrome was measured at 490 nm.

### Measurement of melanin content

The intracellular melanin content was measured as described by Tsuboi *et al.*[24]. The cells were treated with α-MSH (100 nM) for 24 h, and the melanin content was then determined after treatment with either *Lycium chinense* Miller root SFE (2.37, 4.74 and 7.11 mg/mL) or arbutin (2 mM) for an additional 24 h. After treatment, the cell pellets containing a known number of cells were solubilized in 1 N NaOH at 60°C for 60 min. The melanin content was assayed at 405 nm.

### Assay of intracellular tyrosinase activity

The cellular tyrosinase activity was determined as described previously [25]. The cells were treated with α-MSH (100 nM) for 24 h and then with *Lycium chinense* Miller root SFE (2.37, 4.74 and 7.11 mg/mL) or arbutin (2 mM) for 24 h. After the treatments, the cell extracts (100 μL) were mixed with freshly prepared L-DOPA solution (0.1% in PBS) and incubated at 37°C, and the absorbance at 490 nm was measured.

### Western blotting assay

The cells were treated with *Lycium chinense* Miller root SFE (2.37, 4.74 and 7.11 mg/mL) or kojic acid (200 μM) and lysed in proteinase inhibitor containing PBS at 4°C for 20 min. Proteins (50 μg) were resolved by SDS-polyacrylamide gel electrophoresis and electrophoretically transferred to a polyvinylidene fluoride (PVDF) filter. The filter was blocked in 5% fat-free milk in PBST buffer (PBS with 0.05% Tween-20) for 1 h. After a brief wash, the filter was incubated overnight at 4°C with several antibodies; these antibodies included anti- MITF (1:1000), anti-TRP1 (1:6000), anti-TRP2 (1:1000), anti- MC1R (1:500), anti-GAPDH (1:1500), anti-tyrosinase (1:2000), anti-p-p38 (1:500), anti-p38 (1:500), anti-p-JNK (1:500), anti-JNK (1:500), anti-p-ERK (1:500) and anti-ERK (1:500). Following incubation, the filter was extensively washed in PBST buffer. Subsequent incubation with goat anti-mouse antibody (1:10000) conjugated with horseradish peroxidase was conducted at room temperature for 2 h. The blot was visualized using an ECL reagent. The relative amounts of expressed proteins compared to total GAPDH were analyzed using Multi Gauge 3.0 software (Fuji, Tokyo).

### Protein kinase regulators assay

The cells were treated with α-MSH (100 nM) for 24 h followed by a 1-h addition of 10 μM of different protein kinase regulators, including PD98059, SB203580, SP600125 and IBMX. After these treatments, *Lycium chinense* Miller root SFE (2.37, 4.74 and 7.11 mg/mL) and 10 μM of the above-mentioned kinase regulators were added to the cells and incubated for an additional 23 h. The melanin contents were assayed as described above.

### ABTS^+^ scavenging capacity assay

ABTS decolorization assays were carried out as previously described [26], which involved the generation of ABTS^+^ chromophore by the oxidation of ABTS with potassium persulfate. The ABTS radical cation (ABTS^+^) was produced by reacting 7 mM stock solution of ABTS with 2.45 mM potassium persulfate and allowing the mixture to stand in the dark for at least 6 h at room temperature before use. The absorbance at 734 nm was measured 10 min after mixing different concentrations of the *Lycium chinense* Miller root SFE (final concentration 2.37, 4.74, 7.11 mg/mL) with 1 ml of ABTS^+^ solution. The ABTS^+^ scavenging capacity of the extract was compared with that of vitamin C (50 μM) and BHA (0.1 mg/mL).

### Determination of total phenolic content

The amount of total phenolics in the *Lycium chinense* Miller root SFE was determined with the Folin-Ciocalteu reagent [27]. First, a standard curve was plotted using gallic acid as a positive standard. Different concentrations of the root extracts were prepared in 80% methanol. One hundred microliters of sample was dissolved in 500 μL (1/10 dilution) of the Folin-Ciocalteu reagent and 1000 μL of distilled water. The solutions were mixed and incubated at room temperature for 1 min. After 1 min, 1500 μL of 20% sodium carbonate solution was added. The final mixture was shaken and then incubated for 2 h in the dark at room temperature. The absorbances of samples and gallic acid (2.5 and 5 μg/ml) were measured at 760 nm.

### Determination of cellular ROS level

The cells were treated with *Lycium chinense* Miller root SFE (2.37, 4.74 and 7.11 mg/mL) and cultured in 24-well plates for 24 h. The cells were then incubated with 24 mM H_2_O_2_ at 37°C for 30 min. After incubation, 2′,7′-dichlorofluorescein diacetate (DCFH-DA) was added to the wells and cultured for 30 min. The fluorescence intensities of DCF were measured at an excitation wavelength of 504 nm and an emission wavelength of 524 nm [28] using a Fluoroskan Ascent fluorescent reader (Thermo Scientific, Vantaa, Finland). The data were analyzed using Ascent software (Thermo Scientific, Vantaa, Finland).

### Statistical analysis

Statistical analysis of the experimental data points was performed by the ANOVA test, which was used to compare measured data using the SPSS 12.0 statistical software program (SPSS INC. Chicago, USA). Differences were considered statistically significant at *p* < 0.05.

## Results

**Figure 1:**
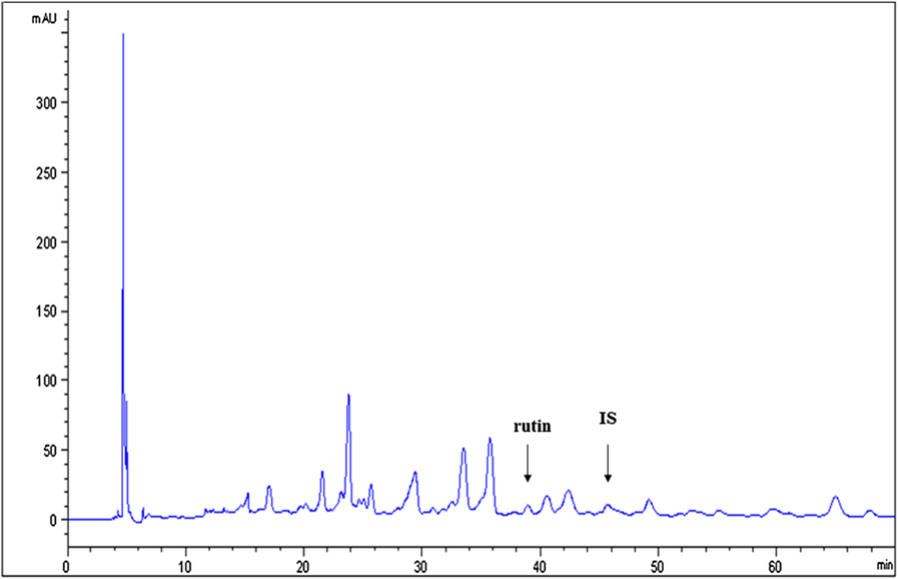
**HPLC chromatogram of *Lycium chinense *** **Miller root SFE.**

HPLC calibration curves were prepared by plotting the peak-area ratios (using naringin as an internal standard) against the corresponding concentrations. For rutin, y = 129.15x - 0.1755 (R^2^ = 0.9921); for liquiritigenin, y = 66.785x + 0.0688 (R^2^ = 0.9906). The detection limits (S/N = 3) for the components were 0.155 (rutin), and 0.387 (liquiritigenin) μg/ml. Suitable amounts of marker substances (rutin, 9.94 μg/mL, and liquiritigenin, 8.29 μg/mL) were added to a sample containing a known content, and the mixture was analyzed by the proposed method. The recoveries of the components were 100.41 (rutin) and 100.43% (liquiritigenin). By substituting the peak-area ratios of the individual peaks for y in the equations, the contents of the individual components in sample extracts were determined by HPLC. The average amount of rutin in *Lycium chinense* Miller root SFE was 23.04 ± 0.172 μg/mL (Figure 1).

**Figure 2:**
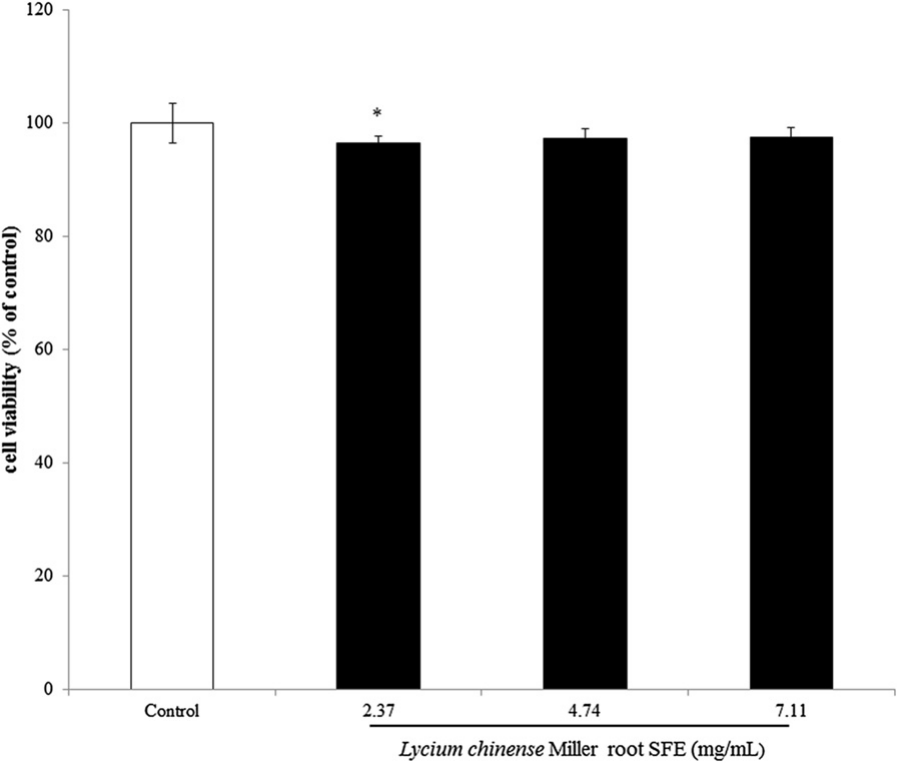
**Effect of *Lycium chinense *Miller root SFE on the proliferation of B16F10 cells.** Cell viability was measured by trypan blue dye exclusion method after 24 h of incubation. Data are expressed as a percentage of the number of viable cells observed with the control, and in each column, mean values ± SD from two or three independent experiments performed in triplicate is presented. *indicates *p* < 0.05 compared to control by ANOVA test.

**Figure 3:**
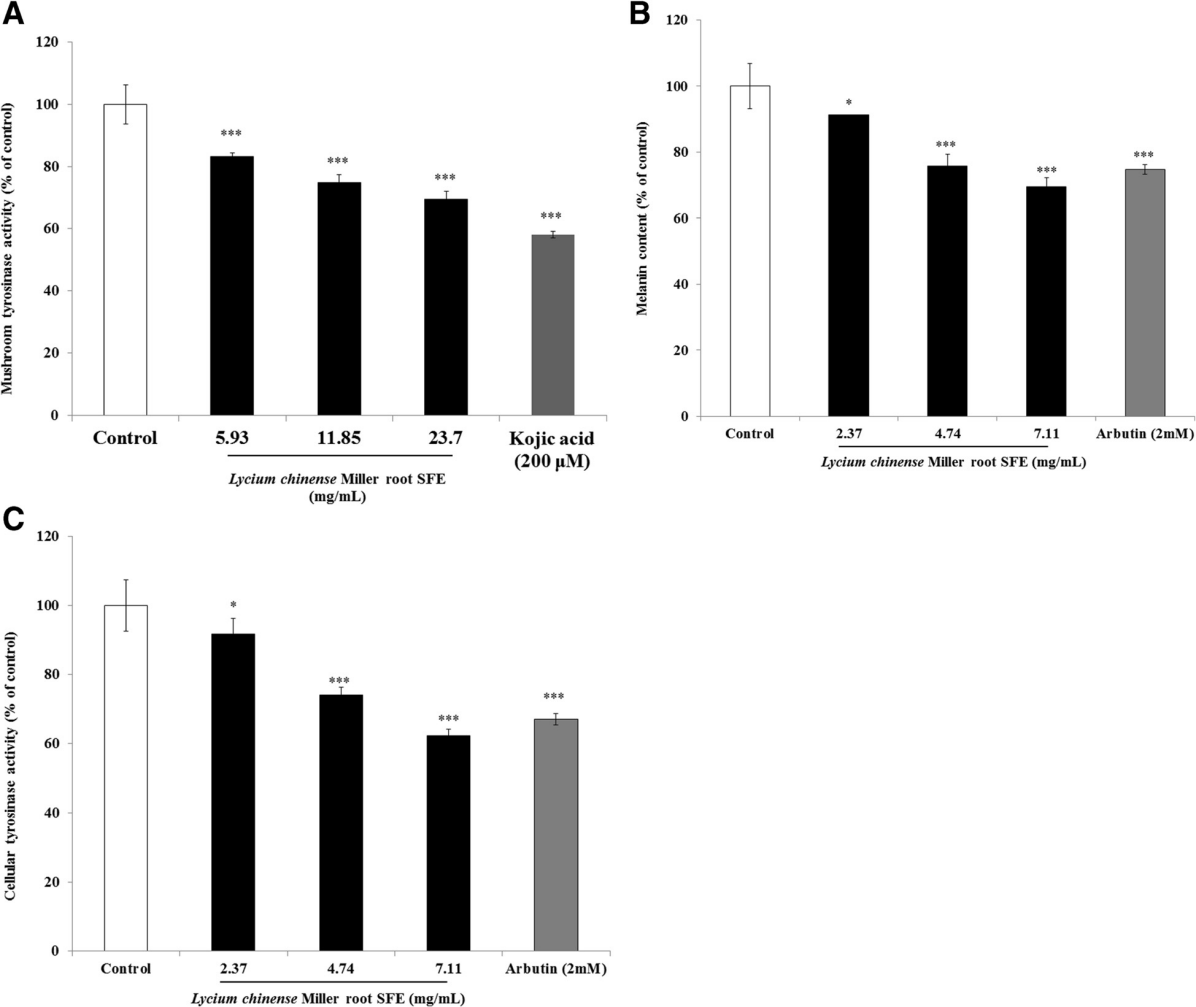
**The inhibitory effects of *Lycium chinense *Miller root SFE on melanogenesis. (A)**. The effects of *Lycium chinense* Miller root SFE on mushroom tyrosinase activity. **(B)**. The effects of *Lycium chinense* Miller root SFE on melanin content in B16F10 cells. **(C)**. The effects of *Lycium chinense* Miller root SFE on tyrosinase activity in B16F10 cells. The results are presented as percentages of the control values, and the data are presented as the mean ± S.D. of three separate experiments. The values are significantly different compared with the control. *p < 0.05; ***p < 0.001.

The MTT assay was used to assess the effect of *Lycium chinense* Miller root SFE on the viability of B16F10 cells. The cells were treated with various concentrations of the root SFE (2.37, 4.74 and 7.11 mg/mL) for 24 h, and then the MTT assay was performed. The results are expressed as percent viability relative to the viability of the control. After treatment, *Lycium chinense* Miller root SFE showed no cytotoxic effect on B16F10 cell viability (Figure 2). The results shown in Figure 3A indicate that the remaining mushroom tyrosinase activity was 83.15 ± 1.25%, 74.84 ± 2.62% and 69.42 ± 2.63% that of the control for the 5.93, 11.85 and 23.7 mg/mL of *Lycium chinense* Miller root SFE treatments, respectively. In addition, the tyrosinase activity was also inhibited by kojic acid (200 μM), and the remaining enzyme activity was 58.14 ± 1.05% that of the control (Figure 3A). Thus, *Lycium chinense* Miller root SFE could be an inhibitor of mushroom tyrosinase, and the IC_50_ was 49.32 mg/mL.

The results indicate that lower concentrations of the *Lycium chinense* Miller root SFE significantly decreased the melanin content in B16F10 melanoma cells. After treatment, the melanin contents in the cells were 91.21 ± 0.18%, 75.81 ± 3.56% and 69.43 ± 2.82% for the 2.37, 4.74 and 7.11 mg/mL of *Lycium chinense* Miller root SFE treatments, respectively. The IC_50_ was 11.01 mg/mL. For the positive standard arbutin (2 mM), the remaining intracellular melanin content was 74.73 ± 1.51% that of the control (Figure 3B). The remaining intracellular tyrosinase activities were 91.69 ± 4.59%, 74.12 ± 2.2% and 62.34 ± 1.8% for the 2.37, 4.74 and 7.11 mg/mL of *Lycium chinense* Miller root SFE treatments, respectively. The IC_50_ of the root SFE was 8.95 mg/mL. The remaining intracellular tyrosinase activity was 67.07 ± 1.6% that of the control after the cells were treated with arbutin (2 mM) (Figure 3C). The results indicate that a higher concentration of *Lycium chinense* Miller root SFE exhibited a potent inhibitory effect on α-MSH-induced tyrosinase activity in B16F10 cells.

**Figure 4:**
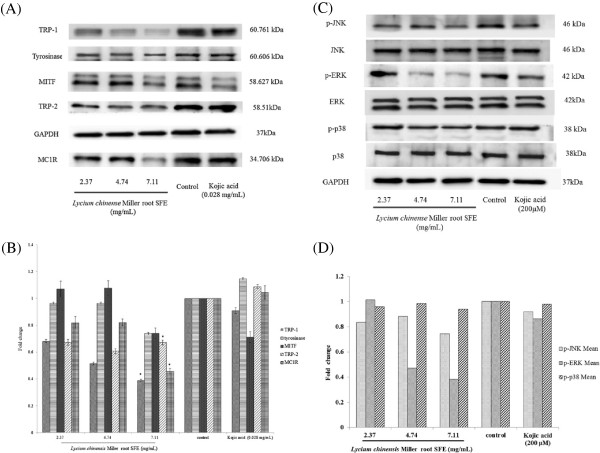
**The effects of *Lycium chinense *Miller root SFE on melanogenesis-related protein expression and signaling pathways. (A)**, **(C)**. Western blotting of cellular proteins in B16F10 cells. **(B)**, **(D)**. The relative amounts of MITF, TRP-1, tyrosinase, TRP-2 and MC1R or phosphorylated proteins (p-JNK, p-ERK and p-p38) compared to the total GAPDH were calculated and analyzed using Multi Gauge 3.0 software, and the values represent the mean of triplicate experiments ± standard deviations. *p < 0.05.

The expression levels of melanogenesis-related proteins were examined using Western blots (Figure 4A). The results indicate that the 2.37-7.11 mg/mL of *Lycium chinense* Miller root SFE treatment led to a reduced level of MC1R, TRP-1 and TRP-2. The inhibitory effects of the root SFE on MITF and tyrosinase expression were apparent at the concentration of 7.11 mg/mL. The fold changes of protein expression levels for MCIR were 0.82, 0.82 and 0.45; 0.68, 0.51 and 0.38 for TRP-1; and 0.67, 0.61 and 0.60 for TRP-2 for the 2.37, 4.74 and 7.11 mg/mL of *Lycium chinense* Miller root SFE treatments, respectively. Additionally, the fold changes of MITF and tyrosinase expressions were 0.74 and 0.73 after treatment with 7.11 mg/mL of the root SFE (Figure 4B).

**Figure 5:**
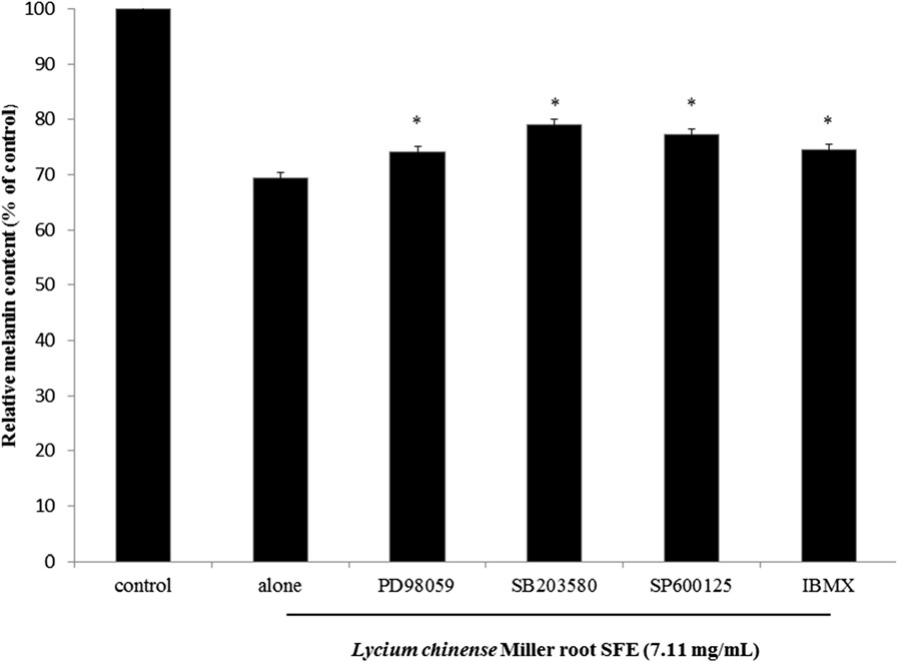
**The effects of *Lycium chinense *Miller root SFE on melanin content in PD98059, SB 203580-, SP600125- and IBMX-treated B16F10 cells.** The results are presented as percentages of the control values, and the data are presented as the mean ± S.D. of three separate experiments. The values are significantly different compared with the control. *p < 0.05.

The JNK signaling pathway is involved in regulating melanogenesis. The results shown in Figure 4C reveal that *Lycium chinense* Miller root SFE decreased the expression of p-JNK; the fold changes of p-JNK in B16F10 cells were 0.83, 0.87 and 0.74 for the 2.37, 4.74 and 7.11 mg/mL of *Lycium chinense* Miller root SFE treatments, respectively. As shown in Figure 4C, various concentrations of *Lycium chinense* Miller root SFE decreased the expression of p-p38; the fold changes of p-p38 in B16F10 cells were 0.95, 0.98 and 0.93 for the 2.37, 4.74 and 7.11 mg/mL of *Lycium chinense* Miller root SFE treatments, respectively. The ERK signaling pathway is also reported to be involved in regulating melanogenesis. The results shown in Figure 4C reveal that *Lycium chinense* Miller root SFE decreased the expression of p-ERK; the fold changes of p-ERK in B16F10 cells were 1.01, 0.46 and 0.37 for the 2.37, 4.74 and 7.11 mg/mL of *Lycium chinense* Miller root SFE treatments, respectively. Furthermore, the addition of the root SFE to SP600125-treated B16F10 cells significantly decreased the cellular melanin content, which indicates that the JNK-mediated signaling pathway was affected by *Lycium chinense* Miller root SFE (Figure 5). To further investigate the role of p38 MAPK signaling on the *Lycium chinense* Miller root SFE-induced anti-melanogenic effect, we employed a specific inhibitor of p38, SB203580, which blocks p38 MAPK signaling. The results shown in Figure 5 reveal that the specific inhibitor of p38 MAPK, SB203580, attenuated α-MSH-stimulated melanin synthesis. These results suggest that *Lycium chinense* Miller root SFE inhibited melanin synthesis by down-regulating p38 MAPK signaling and subsequently decreased melanin synthesis in α-MSH-stimulated B16F10 cells. The addition of *Lycium chinense* Miller root SFE in PD98059-treated B16F10 cells significantly decreased the cellular melanin content. The results indicate that the ERK-mediated signaling pathway involved in melanin production was affected by *Lycium chinense* Miller root SFE treatment (Figure 5). The PKA signaling pathway is associated with regulating melanogenesis. The application of *Lycium chinense* Miller root SFE in IBMX-treated B16F10 cells significantly decreased the cellular melanin content. The results indicate that cAMP-mediated PKA signaling was affected by *Lycium chinense* Miller root SFE (Figure 5).

**Figure 6:**
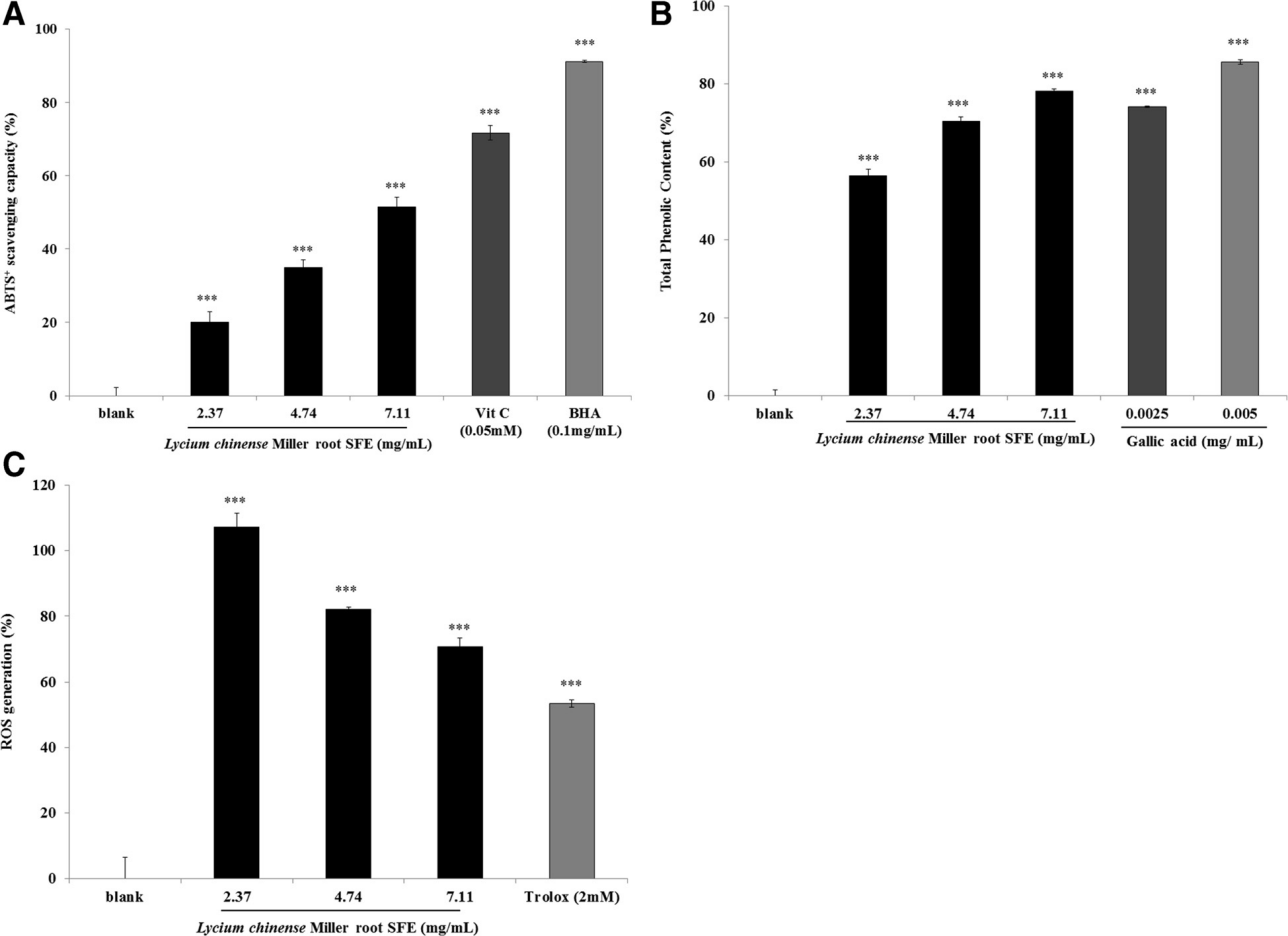
**The antioxidant characteristics of the effect of *Lycium chinense *Miller root SFE. (A)**. ABTS^+^ radical scavenging activity assay. The root extract (final concentration 2.37, 4.74, 7.11 mg/mL), vitamin C (50 μM) and BHA (0.1 mg/mL) were incubated with ABTS^+^ solution. **(B)**. Determination of total phenolic content. Different concentrations of the *Lycium chinense* Miller root SFE (2.37, 4.74, 7.11 mg/mL) and gallic acid (2.5 and 5 μg/ml) were used in the assay. **(C)**. *Lycium chinense* Miller root SFE decreased the cellular ROS level. The B16F10 cells were treated with various concentrations of the root extract (2.37, 4.74, 7.11 mg/mL) or Trolox (2 mM) for 24 h, and then the ROS content was measured by the DCF-DA assay. The results are expressed as percentages of the control values. The data are presented as the mean ± S.D. ***p < 0.001.

The ABTS^+^ assay was employed to measure the antioxidant activity of the *Lycium chinense* Miller root SFE. Different concentrations of the extract (final concentration 2.37, 4.74, 7.11 mg/mL), Vitamin C (50 μM) and BHA (0.1 mg/mL) were incubated with ABTS^+^ solution. The ABTS^+^ scavenging capacities of the extract were 20.12 ± 2.81%, 34.89 ± 2.13% and 51.53 ± 2.65% the activity of the control for extract concentrations of 2.37, 4.74 and 7.11 mg/mL, respectively. Moreover, the ABTS^+^ scavenging capacities of Vitamin C and BHA were 71.72 ± 2.07% and 91.11 ± 0.24%, respectively. The results indicate that the *Lycium chinense* Miller root SFE scavenges ABTS^+^ free radical significantly in a dose-dependent manner. However, the extract showed a lower ABTS^+^ radical scavenging capacity than Vitamin C or BHA does (Figure 6A).

To determine the total phenolic contents of the *Lycium chinense* Miller root SFE (2.37, 4.74 and 7.11 mg/mL), gallic acid (2.5 and 5 μg/ml) was used as a positive standard. The results in Figure 6B showed that the total phenolic contents in 2.37, 4.74 and 7.11 mg/mL of the *Lycium chinense* Miller root SFE was 56.43 ± 1.66%, 70.43 ± 1.15%, 78.15 ± 0.49%, respectively. The phenolic content of 7.11 mg/mL of the extract was similar to that of 2.5 μg/ml of gallic acid (74.2 ± 0.23%).

To confirm the antioxidant capacity of the *Lycium chinense* Miller root SFE in a cellular environment, intracellular ROS levels were evaluated. The concentration of H_2_O_2_ employed was 24 mM. After treatment, the remaining intracellular ROS induced by H_2_O_2_ was 70.62 ± 2.67% for 7.11 mg/mL of the extract and 53.45 ± 1.08% for Trolox (2 mM) (Figure 6C).

## Discussion

The HPLC analysis results shown in Figure 1 reveal that rutin may be the major component in the *Lycium chinense* Miller root SFE. Interestingly, it was reported that the administration of rutin inhibited melanin formation and the decreased melanin content of B16 melanotic melanoma in C57BL/6 mice [29], which supports our proposal that the rutin in the root SFE may play an important role in the inhibition of melanogenesis in melanoma cells.

The MTT assay is a colorimetric assay used to measure cell viability. It can also be used to determine the cytotoxicity of potential medicinal agents and toxic materials because those agents enhance or inhibit cell viability. The results shown in Figure 2 indicate that even higher concentrations of the *Lycium chinense* Miller root SFE (7.11 mg/mL) also showed no cytotoxic effect on B16F10 melanoma cell viability. The *Lycium chinense* Mill root SFE sample (11.85 mg/mL and 23.7 mg/mL) resulted in minor cytotoxicity, while the lower concentration did not substantially induce a cell morphology change 24 h post-irradiation (data not shown). Thus, we applied 2.37-7.11 mg/ml for the following experiments.

Mushroom tyrosinase is widely used as the target enzyme in screening potential inhibitors of melanogenesis. It was first observed that the dosage range (2.37-7.11 mg/mL) of the *Lycium chinense* Miller root SFE could not inhibit the activity of mushroom tyrosinase (data not shown). The *Lycium chinense* Mill root SFE (5.93-23.7 mg/mL) had inhibitory effect on tyrosinase activity using L-DOPA as a substrate. The IC50 of *Lycium chinense* Mill root SFE was found to be 49.32 mg/mL. Kojic acid had strong inhibitory effect on tyrosinase (58.14 ± 1.05% at concentrations of 200 μM) (Figure 3A). Tyrosinase is well known to play an essential role in the first two steps of the melanin synthesis pathway. To elucidate the true inhibitory effect of *Lycium chinense* Miller root SFE on melanin production, the B16F10 melanin content and intracellular tyrosinase activity were determined. The results shown in Figure 3B indicate that the *Lycium chinense* Miller root SFE presents a stronger inhibitory effect on melanin formation than arbutin does. The data provide evidence that *Lycium chinense* Miller root SFE truly blocks melanogenesis in melanoma cells. The results shown in Figure 3C are in accord with the results indicated in Figure 3B, which suggests that the *Lycium chinense* Miller root SFE inhibited intracellular tyrosinase activity and then decreased the melanin content. In those experiments, α-MSH was used as a cAMP inducer to stimulate melanin synthesis. It is reported that α-MSH can bind melanocortin 1 receptor (MC1R) and activate adenylate cyclase, which in turn catalyzes ATP to cAMP and increases intracellular cAMP levels [30]. The results reveal that the *Lycium chinense* Miller root SFE inhibited melanogenesis induced by α-MSH-mediated intracellular cAMP up-regulation.

It has been reported that binding of the human MC1R by its ligands can activate the cAMP signaling pathway and regulate pigmentation of human melanocytes [31]. Melanin biosynthesis in mammalian cells is directly regulated by three major enzymes, tyrosinase, TRP-1 and TRP-2 [32]. Furthermore, MITF is well known to be the most important regulator of melanocyte differentiation and pigmentation and is the major transcriptional regulator of the tyrosinase, TRP-1 and TRP-2 genes [33]. The results shown in Figure 4A and Figure 4B indicate that the *Lycium chinense* Miller root SFE decreased the protein expression levels of those proteins, then inhibited tyrosinase activity and finally decreased the melanin content in the B16F10 cells. The results shown in Figure 4 indicate that *Lycium chinense* Miller root SFE decreased MC1R expression and further suggests that *Lycium chinense* Miller root SFE inhibited melanogenesis induced via α-MSH-mediated intracellular cAMP up-regulation. Moreover, the results shown in Figure 5 further confirm that *Lycium chinense* Miller root SFE inhibited cAMP-mediated PKA signaling.

It has been reported that MAPKs modulate melanin synthesis [34-36]. The MAPK family is composed of three types of protein kinases, extracellular responsive kinase (ERK), c-Jun N-terminal kinase (JNK) and p38 MAPK. The p38 MAPK can activate the cAMP response element-binding protein (CREB), and CREB activates MITF expression, which positively contributes to melanin production [37]. The results in Figure 4C provide evidence that *Lycium chinense* Miller root SFE could inactivate CREB, JNK and p38, in turn inhibiting MITF expression (Figure 4A). Furthermore, Protein kinase A (PKA) signaling is also reported to be involved in melanin production [38]. The α-MSH-mediated elevation of cellular cAMP levels could activate PKA. In turn, activated PKA can activate CREB, leading to the activation of MITF transcriptional activity and resulting in the expressions of melanogenesis-related proteins. Our results shown in Figure 5 also suggest that *Lycium chinense* Miller root SFE inhibits melanin synthesis by blocking the PKA pathway.

The ABTS^+^ free radical has been widely applied to estimate the free radical scavenging activity of antioxidants. Antioxidants can either transfer electrons or hydrogen atoms to ABTS^+^, thus neutralizing the species’ free radical character. In the present study, the *Lycium chinense* Miller root SFE showed lower free radical scavenging activities compared to the activity of Vitamin C or BHA (Figure 6A).

When assaying the total phenolic content of the *Lycium chinense* Miller root SFE, it was interesting to find that a higher concentration of the root extract (7.11 mg/mL) has a highertotal phenolic content. This is probably due to most bioactive compounds such as polyphenols including tannins and flavonoid in the extracts. Polyphenols are one of the major plant compounds with antioxidant activity. The antioxidant activity of phenolic compounds is reported to be mainly due to their redox properties, which can play an important role in adsorbing and neutralizing free radicals, quenching singlet and triplet oxygen or decomposing peroxides [27].

To confirm the antioxidant capacity of *Lycium chinense* Miller root SFE in a cellular environment, intracellular ROS levels were evaluated. The principle of the assay is based on the fact that DCFH-DA diffuses through the cell membrane and is enzymatically hydrolyzed by esterase to DCFH, which reacts with ROS (such as H_2_O_2_) to yield DCF. Rapid increases in DCF indicate the oxidation of DCFH by intracellular radicals. The results reveal that the flower extract depleted intracellular ROS in a dose-dependent manner. The skin is exposed to UV and environmental oxidizing pollutants and is a preferred target of oxidative stress. It is reported that ultraviolet irradiation induces the formation of reactive oxygen species (ROS) in cutaneous tissue, provoking toxic changes such as lipid peroxidation and enzyme inactivation [39]. It is reported that chronic exposure to solar UV radiation plays a role in the initiation of several skin hyperpigmentation disorders [40]. Therefore, there is an increasing need for new and effective agents that exhibit photoprotection and skin anti-hyperpigmentation activities to prevent abnormal skin pigmentation disorders. To date, there is no report about the effect of *Lycium chinense* Miller root SFE on melanin production. This is the first study to evidence the potential inhibitory effect of *Lycium chinense* Miller root SFE on melanogenesis in B16F10 melanoma cells. In addition, the root extract also shows antioxidant capacities, which fits the trend of skin anti-hyperpigmentation agents that show dual functions in anitimelanogenesis and antioxidation. Recently, several plants such as *Paeonia suffruticosa*[41] and chestnut flower extract [42] have also been reported to show antioxidant and antimelanogenic properties similar to those of *Lycium chinense* Miller root SFE. The results suggest that *Lycium chinense* Miller root SFE decreased melanin production due to its depletion of cellular ROS.

Our results indicate that *Lycium chinense* Miller root SFE inhibited melanogenesis in B16F10 cells by down-regulation of both mitogen-activated protein kinases (MAPK) and protein kinase A (PKA) signaling pathways or through its antioxidant properties. Hence, *Lycium chinense* Miller root SFE could be used as an effective skin anti-hyperpigmentation agent.

## Conclusions

This is the first report on the inhibitory effect of *Lycium chinense* Miller root SFE on melanin synthesis. We also analyzed the antioxidant capacities of the root SFE of *Lycium chinense* Miller. The present study concluded that *Lycium chinense* Miller root SFE inhibits melanin synthesis in B16F10 melanoma cells and showed antioxidant potential. The present results indicate that the *Lycium chinense* Miller root SFE inhibited melanin synthesis in B16F10 melanoma cells by down-regulation of both mitogen-activated protein kinases (MAPK) and protein kinase A (PKA) signaling pathways or through its intracellular antioxidant properties. Hence, the SFE of *Lycium chinense* Miller root could be applied as a type of dermatological anti-hyperpigmentation agent in skin care products.

## Competing interests

All authors are in agreement with the content of the manuscript and the authors do not have any actual or potential conflict of interest, including any financial competing interests, non-financial competing interests, personal or other relationships with other people or organizations within that could inappropriately influence (bias) the work.

## Authors’ contributions

HCH carried out the tyrosinase-related studies, participated in the enzyme assays and drafted the manuscript. WY H performed HPLC analysis. WY H carried out antioxidant experiments. TCT, carried out the supercritical fluid CO_2_ extraction (SFE) of *Lycium chinense* Miller root. WPK and KJC carried out Western blot experiments. TMC participated in design and coordination of the study, performed the statistical analysis and drafted the manuscript. All authors read and approved the final manuscript.

## Pre-publication history

The pre-publication history for this paper can be accessed here:

http://www.biomedcentral.com/1472-6882/14/208/prepub

## References

[B1] Spritz RA Hearing VJ Jr Genetic disorders of pigmentation Adv Hum Genet 1994 22 1 45 7539206 10.1007/978-1-4757-9062-7_1

[B2] Briganti S Camera E Picardo M Chemical and instrumental approaches to treat hyperpigmentation Pigment Cell Res 2003 16 2 101 110 12622786 10.1034/j.1600-0749.2003.00029.x

[B3] Funasaka Y Komoto M Ichihashi M Depigmenting Effect of α-Tocopheryl Ferulate on Normal Human Melanocytes Pigment Cell Res 2000 13 170 174 11041377 10.1111/j.0893-5785.2000.130830.x

[B4] Seo S-Y Sharma VK Sharma N Mushroom Tyrosinase: Recent Prospects J Agric Food Chem 2003 51 10 2837 2853 12720364 10.1021/jf020826f

[B5] Hearing VJ Jimenez M Mammalian tyrosinase–the critical regulatory control point in melanocyte pigmentation Int J Biochem 1987 19 12 1141 1147 3125075 10.1016/0020-711x(87)90095-4

[B6] Jimenez-Cervantes C Solano F Kobayashi T Urabe K Hearing VJ Lozano JA Garcia-Borron JC A new enzymatic function in the melanogenic pathway. The 5,6-dihydroxyindole-2-carboxylic acid oxidase activity of tyrosinase-related protein-1 (TRP1) J Biol Chem 1994 269 27 17993 18000 8027058

[B7] Tsukamoto K Jackson IJ Urabe K Montague PM Hearing VJ A second tyrosinase-related protein, TRP-2, is a melanogenic enzyme termed DOPAchrome tautomerase EMBO J 1992 11 2 519 526 1537333 10.1002/j.1460-2075.1992.tb05082.x PMC556482

[B8] Garcia-Borron JC Sanchez-Laorden BL Jimenez-Cervantes C Melanocortin-1 receptor structure and functional regulation Pigment Cell Res 2005 18 6 393 410 16280005 10.1111/j.1600-0749.2005.00278.x

[B9] Yamakoshi J Otsuka F Sano A Tokutake S Saito M Kikuchi M Kubota Y Lightening effect on ultraviolet-induced pigmentation of guinea pig skin by oral administration of a proanthocyanidin-rich extract from grape seeds Pigment Cell Res 2003 16 6 629 638 14629720 10.1046/j.1600-0749.2003.00093.x

[B10] Imokawa G Analysis of initial melanogenesis including tyrosinase transfer and melanosome differentiation through interrupted melanization by glutathione J Invest Dermatol 1989 93 1 100 107 2501395 10.1111/1523-1747.ep12277369

[B11] Kumano Y Sakamoto T Egawa M Iwai I Tanaka M Yamamoto I In vitro and in vivo prolonged biological activities of novel vitamin C derivative, 2-O-alpha-D-glucopyranosyl-L-ascorbic acid (AA-2G), in cosmetic fields J Nutr Sci Vitaminol (Tokyo) 1998 44 3 345 359 9742456 10.3177/jnsv.44.345

[B12] Huang H-C Hsieh W-Y Niu Y-L Chang T-M Inhibition of melanogenesis and antioxidant properties of Magnolia grandiflora L. flower extract BMC Complement Altern Med 2012 12 1 72 22672352 10.1186/1472-6882-12-72 PMC3404006

[B13] Huang HC Wang HF Yih KH Chang LZ Chang TM Dual Bioactivities of Essential Oil Extracted from the Leaves of Artemisia argyi as an Antimelanogenic versus Antioxidant Agent and Chemical Composition Analysis by GC/MS Int J Mol Sci 2012 13 11 14679 14697 23203088 10.3390/ijms131114679 PMC3509604

[B14] Huang HC Chang TY Chang LZ Wang HF Yih KH Hsieh WY Chang TM Inhibition of melanogenesis versus antioxidant properties of essential oil extracted from leaves of Vitex negundo Linn and chemical composition analysis by GC-MS Molecules 2012 17 4 3902 3916 22466851 10.3390/molecules17043902 PMC6268308

[B15] Huang HC Huang WY Chiu SH Ke HJ Chiu SW Wu SY Kuo FS Chang TM Antimelanogenic and antioxidative properties of Bifidobacterium bifidum Arch Dermatol Res 2011 303 7 527 531 21365207 10.1007/s00403-011-1135-y

[B16] Huang HCS Ke HJ Chiu SW Wu SY Chang TM Antimelanogenic and antioxidant activities of Bifidobacterium infantis Infect Immun 2011 5 7

[B17] Kim SY Lee KH Chang KS Bock JY Jung MY Taste and flavor compounds in box thorn (Lycium chinense Miller) leaves Food Chem 1997 58 4 297 303

[B18] Xiao PG Xing ST Wang LW Immunological aspects of Chinese medicinal plants as antiageing drugs J Ethnopharmacol 1993 38 2–3 167 175 8510465 10.1016/0378-8741(93)90012-t

[B19] Kim SY Kim HP Huh H Kim YC Antihepatotoxic zeaxanthins from the fruits ofLycium chinense Arch Pharm Res 1997 20 6 529 532 18982254 10.1007/BF02975206

[B20] Funayama SY K Konno C Hikino H Structure of kukoamine A, a hypotensive principle of Lycium chinense root barks1 Tetrahedron Lett 1980 21 14 2

[B21] Kim HP Kim SY Lee EJ Kim YC Kim YC Zeaxanthin dipalmitate from Lycium chinense has hepatoprotective activity Res Commun Mol Pathol Pharmacol 1997 97 3 301 314 9387190

[B22] Tada H Shiho O Kuroshima K Koyama M Tsukamoto K An improved colorimetric assay for interleukin 2 J Immunol Methods 1986 93 2 157 165 3490518 10.1016/0022-1759(86)90183-3

[B23] Bilodeau ML Greulich JD Hullinger RL Bertolotto C Ballotti R Andrisani OM BMP-2 stimulates tyrosinase gene expression and melanogenesis in differentiated melanocytes Pigment Cell Res 2001 14 5 328 336 11601654 10.1034/j.1600-0749.2001.140504.x

[B24] Tsuboi T Kondoh H Hiratsuka J Mishima Y Enhanced melanogenesis induced by tyrosinase gene-transfer increases boron-uptake and killing effect of boron neutron capture therapy for amelanotic melanoma Pigment Cell Res 1998 11 5 275 282 9877098 10.1111/j.1600-0749.1998.tb00736.x

[B25] Yang JY Koo JH Song YG Kwon KB Lee JH Sohn HS Park BH Jhee EC Park JW Stimulation of melanogenesis by scoparone in B16 melanoma cells Zhongguo Yao Li Xue Bao 2006 27 11 1467 1473 17049123 10.1111/j.1745-7254.2006.00435.x

[B26] Re R Pellegrini N Proteggente A Pannala A Yang M Rice-Evans C Antioxidant activity applying an improved ABTS radical cation decolorization assay Free Radic Biol Med 1999 26 9–10 1231 1237 10381194 10.1016/s0891-5849(98)00315-3

[B27] Galato D Ckless K Susin MF Giacomelli C Ribeiro-do-Valle RM Spinelli A Antioxidant capacity of phenolic and related compounds: correlation among electrochemical, visible spectroscopy methods and structure-antioxidant activity Redox Rep 2001 6 4 243 250 11642715 10.1179/135100001101536391

[B28] Murrant CL Reid MB Detection of reactive oxygen and reactive nitrogen species in skeletal muscle Microsc Res Tech 2001 55 4 236 248 11748862 10.1002/jemt.1173

[B29] Drewa G Schachtschabel DO Palgan K Grzanka A Sujkowska R The influence of rutin on the weight, metastasis and melanin content of B16 melanotic melanoma in C57BL/6 mice Neoplasma 1998 45 4 266 271 9890672

[B30] Busca R Ballotti R Cyclic AMP a key messenger in the regulation of skin pigmentation Pigment Cell Res 2000 13 2 60 69 10841026 10.1034/j.1600-0749.2000.130203.x

[B31] Abdel-Malek Z Swope V Collins C Boissy R Zhao H Nordlund J Contribution of melanogenic proteins to the heterogeneous pigmentation of human melanocytes J Cell Sci 1993 106 Pt 4 1323 1331 8126111 10.1242/jcs.106.4.1323

[B32] Kameyama K Sakai C Kuge S Nishiyama S Tomita Y Ito S Wakamatsu K Hearing VJ The expression of tyrosinase, tyrosinase-related proteins 1 and 2 (TRP1 and TRP2), the silver protein, and a melanogenic inhibitor in human melanoma cells of differing melanogenic activities Pigment Cell Res 1995 8 2 97 104 7659683 10.1111/j.1600-0749.1995.tb00648.x

[B33] Levy C Khaled M Fisher DE MITF: master regulator of melanocyte development and melanoma oncogene Trends Mol Med 2006 12 9 406 414 16899407 10.1016/j.molmed.2006.07.008

[B34] Hirata N Naruto S Ohguchi K Akao Y Nozawa Y Iinuma M Matsuda H Mechanism of the melanogenesis stimulation activity of (-)-cubebin in murine B16 melanoma cells Bioorg Med Chem 2007 15 14 4897 4902 17521910 10.1016/j.bmc.2007.04.046

[B35] Kim DS Jeong YM Park IK Hahn HG Lee HK Kwon SB Jeong JH Yang SJ Sohn UD Park KC A new 2-imino-1,3-thiazoline derivative, KHG22394, inhibits melanin synthesis in mouse B16 melanoma cells Biol Pharm Bull 2007 30 1 180 183 17202683 10.1248/bpb.30.180

[B36] Smalley K Eisen T The involvement of p38 mitogen-activated protein kinase in the alpha-melanocyte stimulating hormone (alpha-MSH)-induced melanogenic and anti-proliferative effects in B16 murine melanoma cells FEBS Lett 2000 476 3 198 202 10913613 10.1016/s0014-5793(00)01726-9

[B37] Singh SK Sarkar C Mallick S Saha B Bera R Bhadra R Human placental lipid induces melanogenesis through p38 MAPK in B16F10 mouse melanoma Pigment Cell Res 2005 18 2 113 121 15760340 10.1111/j.1600-0749.2005.00219.x

[B38] Hirobe T Basic fibroblast growth factor stimulates the sustained proliferation of mouse epidermal melanoblasts in a serum-free medium in the presence of dibutyryl cyclic AMP and keratinocytes Development 1992 114 2 435 445 1317290 10.1242/dev.114.2.435

[B39] Emerit I Free radicals and aging of the skin EXS 1992 62 328 341 1450595 10.1007/978-3-0348-7460-1_33

[B40] Mukhtar H Elmets CA Photocarcinogenesis: mechanisms, models and human health implications Photochem Photobiol 1996 63 4 356 357 8934734 10.1111/j.1751-1097.1996.tb03040.x

[B41] Ding HY Chou TH Lin RJ Chan LP Wang GH Liang CH Antioxidant and antimelanogenic behaviors of Paeonia suffruticosa Plant Foods Hum Nutr 2011 66 3 275 284 21656165 10.1007/s11130-011-0235-3

[B42] Sapkota K Park SE Kim JE Kim S Choi HS Chun HS Kim SJ Antioxidant and antimelanogenic properties of chestnut flower extract Biosci Biotechnol Biochem 2010 74 8 1527 1533 20699587 10.1271/bbb.100058

